# Short-term in vivo testing to discriminate genotoxic carcinogens from non-genotoxic carcinogens and non-carcinogens using next-generation RNA sequencing, DNA microarray, and qPCR

**DOI:** 10.1186/s41021-023-00262-9

**Published:** 2023-02-09

**Authors:** Chie Furihata, Takayoshi Suzuki

**Affiliations:** 1 Division of Molecular Target and Gene Therapy Products, National Institute of Health Sciences, 3-25-26 Tonomachi, Kawasaki-Ku, Kawasaki, Kanagawa 210-9501 Japan; 2 School of Science and Engineering, Aoyama Gakuin University, Sagamihara, Kanagawa 252-5258 Japan

**Keywords:** RNA-Seq, DNA microarray, qPCR, Rodent short-term in vivo test, Genotoxic carcinogen, Non-genotoxic carcinogen, Non-carcinogen

## Abstract

Next-generation RNA sequencing (RNA-Seq) has identified more differentially expressed protein-coding genes (DEGs) and provided a wider quantitative range of expression level changes than conventional DNA microarrays. JEMS·MMS·Toxicogenomics group studied DEGs with targeted RNA-Seq on freshly frozen rat liver tissues and on formalin-fixed paraffin-embedded (FFPE) rat liver tissues after 28 days of treatment with chemicals and quantitative real-time PCR (qPCR) on rat and mouse liver tissues after 4 to 48 h treatment with chemicals and analyzed by principal component analysis (PCA) as statics. Analysis of rat public DNA microarray data (Open TG-GATEs) was also performed. In total, 35 chemicals were analyzed [15 genotoxic hepatocarcinogens (GTHCs), 9 non-genotoxic hepatocarcinogens (NGTHCs), and 11 non-genotoxic non-hepatocarcinogens (NGTNHCs)]. As a result, 12 marker genes (*Aen*, *Bax*, *Btg2*, *Ccnf*, *Ccng1*, *Cdkn1a*, *Gdf15*, *Lrp1*, *Mbd1*, *Phlda3*, *Plk2*, and *Tubb4b*) were proposed to discriminate GTHCs from NGTHCs and NGTNHCs. U.S. Environmental Protection Agency studied DEGs induced by 4 known GTHCs in rat liver using DNA microarray and proposed 7 biomarker genes, *Bax*, *Bcmp1*, *Btg2*, *Ccng1*, *Cdkn1a*, *Cgr19*, and *Mgmt* for GTHCs. Studies involving the use of whole-transcriptome RNA-Seq upon exposure to chemical carcinogens in vivo have also been performed in rodent liver, kidney, lung, colon, and other organs, although discrimination of GTHCs from NGTHCs was not examined. Candidate genes published using RNA-Seq, qPCR, and DNA microarray will be useful for the future development of short-term in vivo studies of environmental carcinogens using RNA-Seq.

**Supplementary Information:**

The online version contains supplementary material available at 10.1186/s41021-023-00262-9.

## Background

Lovett published the article “Toxicogenomics: Toxicologists brace for genomics revolution” in Science in 2000. He described the new approach of toxicogenomics, in which DNA microarrays are used to profile gene expression in cells exposed to test compounds [1]. Quantitative real-time PCR (qPCR) has been used independently or to confirm DNA microarray results [2, 3]. However, RNA-Seq is now an important tool for examining the role of the transcriptome in biological processes [4], which could surpass DNA microarray and qPCR [5, 6]. Nevertheless, to date, only a small number of studies have been published on the discrimination of GTHCs from NGTHCs and NGTNHCs using RNA-Seq-based toxicogenomics [5–31] (File 1).

Carcinogenicity testing plays an essential role in identifying carcinogens in environmental chemistry and pharmaceutical drug development. However, it is a time-consuming and labor-intensive process to evaluate the carcinogenicity with conventional 2-year rodent-based animal studies [32]. There is thus an increased need to develop novel alternative approaches to these rodent bioassays for assessing the carcinogenicity of substances [33].

Carcinogens have conventionally been divided into two categories according to their presumed mode of action: genotoxic carcinogens (GTCs) and non-genotoxic carcinogens (NGTCs). An OECD expert group defined that a GTC has the potential to induce cancer by interacting directly with DNA and/or the cellular apparatus involved in preserving the integrity of the genome, while an NGTC has the potential to induce cancer without interacting directly with either DNA or the above-mentioned apparatus [34].

Bevan and Harrison asserted that genotoxic carcinogens are usually identified based on positive results in different in vitro and in vivo test systems, including detection of DNA strand breaks, unscheduled DNA synthesis, sister chromatid exchange, DNA adduct formation, mitotic recombination, and gene mutation. Typical tests of mutagenicity include the Ames test, in vitro metaphase chromosome aberration assay, in vitro micronucleus assay, and mouse lymphoma L5178Y cell Tk (thymidine kinase) gene mutation assay, in vivo micronucleus assay in rodents, and transgenic rodent mutation assay. NGTCs are considered to have a threshold for exerting hazardous effects and guidelines regarding appropriate levels of exposure to them are set by the various authoritative bodies in the same way as for other hazardous substances. Bevan and Harrison recommend that clear differentiation between threshold and non-threshold carcinogens should be made by all expert groups and regulatory bodies dealing with carcinogen classification and risk assessment [35].

RNA-Seq has identified more DEGs and provided a wider quantitative range of expression level changes than conventional DNA microarrays. Because of its wider dynamic range as well as its ability to identify a larger number of DEGs, RNA-Seq may generate more insight into mechanisms of toxicity and mode of action (MOA) [6]. In this context, the successful development of a short-term in vivo assay in rodents for discriminating GTCs, NGTCs, and non-carcinogens (NCs) using RNA-Seq would be valuable.

Only a few papers have been published on discriminating GTCs from NGTCs using RNA-Seq in vivo [8, 9]. Therefore, this review also includes data on discriminating GTCs, NGTCs, and NHCs using DNA microarray and qPCR [36–47], as these data would be helpful in creating a toxicogenomics database. This review also incorporates recent reports on whole-transcriptome RNA-Seq on animals in vivo, in the liver, kidney, and other organs, although reports did not include the discrimination of GTCs from NGTCs [5–31].

In this manuscript, we introduce candidate marker genes published using RNA-Seq, qPCR, and DNA microarray to develop RNA-Seq to discriminate GTCs, NGTCs, and NCs among the chemicals to which humans are exposed in daily life.

### Discrimination of GTHCs and NGTHCs and/or NGTNHCs using DNA microarray and qPCR in vivo

In the early days of toxicogenomics research, Ellinger-Ziegelbauer et al. reported DEGs in rat liver upon exposure to 4 GTHCs [ 2-nitrofluorene (2NF), dimethylnitrosamine (DMN), 4-(methylnitrosamino)-1-(3-pyridyl)-1-butanone (NNK), and aflatoxin B1 (AFB1)] and 4 NGTHCs [methapyrilene (MPy), diethylstilbestrol (DES), Wy-14643, and piperonylbutoxide (PBO)] for 1–14 days using DNA microarray and the support vector machine (SVM) algorithm as a statistical analysis [36–38]. They presented marker genes, such as *Cdkn1a*, *Ccng1*, and *Mgmt* for GTHCs and *Apex1*, *Pcna*, *Cdk1*, *Ccnb1*, *Rps27*, *Hspd1*, and *Hspa9* for NGTHCs, whose expression was characteristically changed upon exposure to these carcinogens [36].

**Fig. 1:**
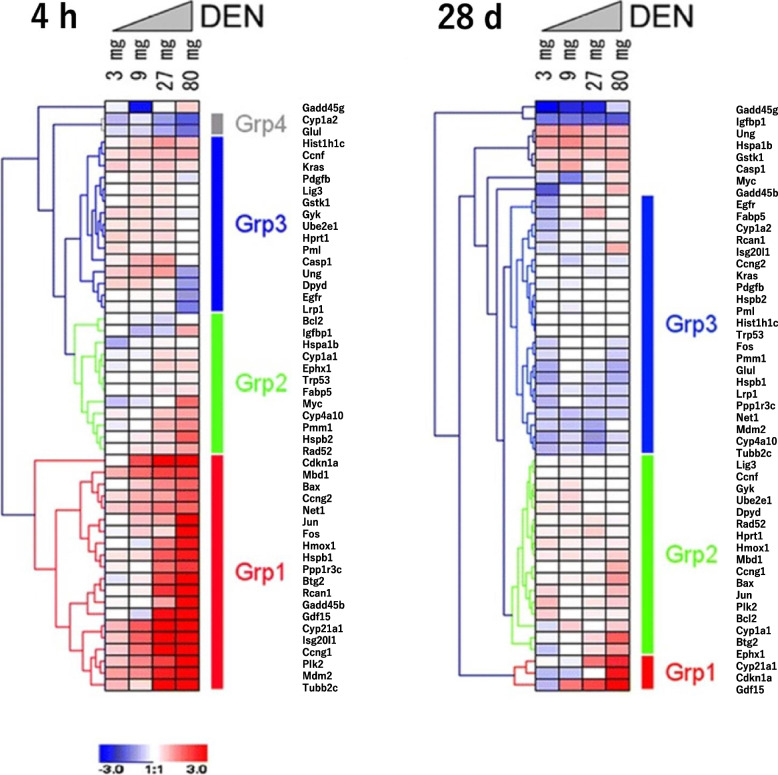
Cluster analysis of gene expression in mouse liver after DEN treatment quantified by qPCR. The expression of 50 genes was clustered by hierarchical clustering after DEN treatment. Results of 4 h and 28 days after a single shot were analyzed separately. The color displays show the log2 (expression ratio) as (1) red when the treatment sample is up-regulated relative to the control sample, (2) blue when the treatment sample is down-regulated relative to the control sample, and (3) white when the log2 (expression ratio) is close to zero [40]. At 4 h, all 20 Grp 1 genes showed a dose-dependent increase of more than 3–64-fold. Twelve Grp 2 genes were suggested to have a gradual dose-dependent increase of less than that for the expression in Grp1. Two Grp 4 genes exhibited a dose-dependent decrease of less than 0.3-fold. Fifteen Grp 3 genes showed few changes in gene expression. At 28 days, three Grp 1 genes showed a dose-dependent increase of more than four-fold. Seventeen Grp 2 genes were suggested to have a gradual dose-dependent increase, though less than that for the expression in Grp 1. Ungrouped Igfbp1 showed a dose-dependent decrease of less than 0.3-fold. 22 Grp 3 genes showed fewer changes in gene expression

**Fig. 2:**
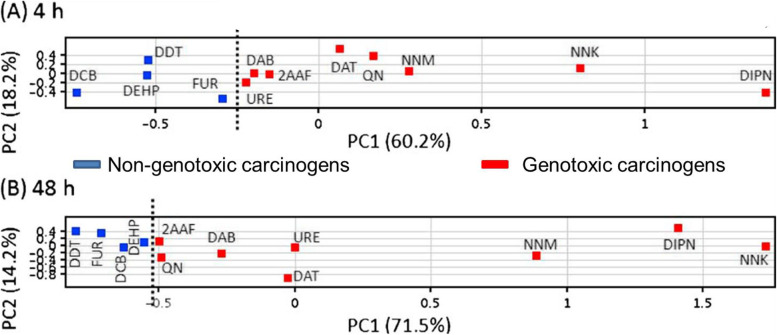
Principal component analysis (PCA) of the gene expression levels in mouse liver after a single shot between genotoxic and non-genotoxic hepatocarcinogens as quantified by qPCR. **A** 4 h with 7 genes (*Btg2*, *Ccnf*, *Ccng1*, *Lrp1*, *Mbd1*, *Phlda3*, and *Tubb2c*), **B** 48 h with 12 genes (*Aen*, *Bax*, *Btg2*, *Ccnf*, *Ccng1*, *Cdkn1a*, *Gdf15*, *Lrp1*, *Mbd1*, *Phlda3*, *Plk2,* and *Tubb2c*). GTHCs (red-colored, DIPN: diisopropanolnitrosamine, NNK: 4-(methylnitrosamino)-1-(3-pyridyl)-1-butanone, NNM: *N*-nitrosomorpholine, QN: quinoline, DAT: 2,4-diaminotoluene, DAB: 4-dimethylaminoazobenzene, 2AAF: 2-acetylaminofluorene, URE: urethane) and NGTHCs (bleu-colored, FUR: furan, DDT: dichlorodiphenyltrichloroethane, DEHP: di(2-ethylhexyl)phthalate, DCB: 1,4-dichlorobenzene). A dashed line is added between genotoxic and non-genotoxic hepatocarcinogens [41]

**Fig. 3:**
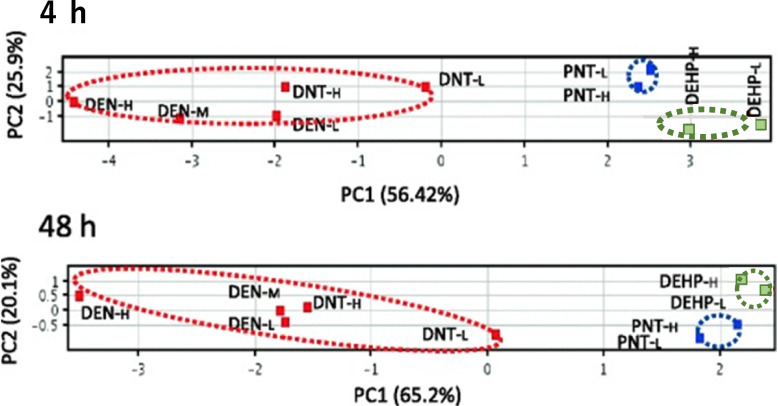
Principal component analysis (PCA) of the gene expression levels under treatment with 3 types of carcinogens in rat liver as quantified by qPCR. GTHCs (red-colored, DEN-L: DEN low dose, DEN-M: DEN middle dose, DEN-H: DEN high dose, DNT-L: DNT low dose and DNT-H: DNT high dose), an NGTHC (green-colored, DEHP-L: DEHP low dose and DEHP-H: DEHP high dose), and an NGTNHC (blue-colored, PNT-L: PNT low dose and PNT-H: PNT high dose). **A** 4 h, with 16 genes (*Ccnf*, *Ccng1*, *Cyp4a1*, *Ddit4l*, *Egfr*, *Gadd45g*, *Gdf15*, *Hspb1*, *Ighbp1*, *Jun*, *Myc*, *Net1*, *Phlda3*, *Pml*, *Rcan1*, and *Tubb2c*), **B** 48 h, with 10 genes (Aen, *Ccng1*, *Cdkn1a*, *Cyp21a1*, *Cyp4a1*, *Gdf15*, *Igfbp1*, *Mdm2*, *Phlda3,* and *Pmm1*). PCA successfully differentiated GTHCs (red circle) from an NGTHC (green circle) and an NGTNHC (blue circle) with principal component 1 at 4 and 48 h [43]

In the form of collaborative studies of the Toxicogenomics/The Japanese Environmental Mutagen Society ·Mammalian Mutagenicity Study Group (JEMS·MMS), Furihata et al. conducted research to discriminate GTHCs from NGTHCs and/or NGTNHCs using rodent liver [3, 39–43]. They selected 50 candidate marker genes and *Gapdh* as a control gene for normalization based on their preliminary results with nine chemicals using an original DNA microarray and Affymetrix GeneChip Mu74AV2. They reported the dose-dependent changes of expression determined by qPCR at 4 h and 28 days for 50 genes in the liver of mice treated with a single dose of two *N*-nitroso GTHCs, diethylnitrosamine (DEN) and ethylnitrosourea (ENU), as shown in Fig. 1 [40]. Next, they studied the effects of 12 chemicals on mouse liver at 4 and 48 h after their single dosing and successfully discriminated eight GTHCs [2-acetylaminofluorene (2AAF), 2,4-diaminotoluene, diisopropanolnitrosamine, 4-dimethylaminoazobenzene, NNK, *N*-nitrosomorpholine, quinoline, and urethane] from four NGTHCs [1,4-dichlorobenzene, dichlorodiphenyltrichloroethane, di(2-ethylhexyl)phthalate (DEHP), and furan] using qPCR and PCA, as shown in Fig. 2 [41]. They also identified by qPCR that 4 and 48 h after administration were key time points from the time-dependent changes in gene expression during the acute phase (4 to 48 h) following the administration of chrysene [42]. Additionally, in rat liver, they successfully discriminated two GTHCs (DEN and 2,6-dinitrotoluene) from an NGTHC (DEHP) and an NGTNHC (phenacetin) at 4 and 48 h, as shown in Fig. 3 [43]. They then proposed 12 candidate marker genes (*Aen*, *Bax*, *Btg2*, *Ccnf*, *Ccng1*, *Cdkn1a*, *Gdf15*, *Lrp1*, *Mbd1*, *Phlda3*, *Plk2*, and *Tubb4b*) (JEMS/MMS marker genes) to discriminate GTHCs and NGTHCs and/or NGTNHCs. Subsequent gene pathway analysis on these genes by Ingenuity Pathway Analysis indicated that they are particularly involved in the DNA damage response, resulting from the signal transduction of a p53-class mediator leading to the induction of apoptosis. These studies suggest that the application of PCA to the gene expression profile in rodent liver during the acute phase is useful for predicting that a chemical is a GTHC rather than an NGTHC and/or an NGTNHC [41, 43].

U.S. Environmental Protection Agency (EPA) studied DEGs induced by 4 known GTHCs: 2NF, AFB1, NNK, and DMN in rat liver and proposed 7 biomarker genes, *Bax*, *Bcmp1*, *Btg2*, *Ccng1*, *Cdkn1a*, *Cgr19*, and *Mgmt* for GTHCs [44]. Four genes, *Bax*, *Btg2*, *Ccng1,* and *Cdkn1a* were also proposed as GTHC-associated DEGs by Furihata et al. [41, 43].

Park et al. studied DEGs induced by 2 GTHCs (2AAF and DEN), 1 GTC, melphalan, and 1 NGTNC, 1-naphthylisothiocynate in rasH2 mouse liver upon repeated administrations for 7- and 91- days using DNA microarray and qPCR and presented the results in a heatmap. They selected 68 significantly deregulated genes that represented a GTHC-specific signature; these genes were commonly deregulated in both the 2AAF- and DEN-treated rasH2 mice, namely, 52 up-regulated genes, including *Aen*, *Bax*, *Btg2*, *Ccng1*, *Cdkn1a*, *Ddit4l*, *Plk2*, *Mdm2*, *Phlda3*, and *Tubb4b* as also proposed as GTHC-associated DEGs upon exposure to DEN and 2AAF by Furihata et al. [41, 43], and 16 down-regulated genes, [45].

Kossler et al. examined a total of 13 chemicals, including 3 known GTHCs: (C.I. Direct Black 38, DMN, and 4,4’-methylenedianiline), 3 NGTHCs: (1,4-dichlorobenzene, phenobarbital sodium, and piperonyl butoxide), 4 NHCs (medical drugs;): cefuroxime sodium, nifedipine, prazosin hydrochloride, and propranolol hydrochloride), and 3 chemicals exhibiting ambiguous results in genotoxicity testing: (cyproterone acetate, thioacetamide, and Wy-14643), in CD-1 mouse liver after their oral administration for 3 and 14 days. They proposed 51 marker candidate genes for differentiating GTHCs from NGTHCs and NHCs **(**Table 1**)** and 58 marker candidate genes for differentiating NGTHCs from GTHCs and NHCs **(**Table 2**)** in mouse liver, as examined with DNA microarray, in the course of the IMI MARCAR (Innovative Medicines Initiative/Biomarkers and molecular tumor classification for non-genotoxic carcinogenesis) project, involving a European consortium of partners in EFPIA “a research-based pharmaceutical industry operation in Europe” and academics [46]. Using two-step heatmaps, they suggested successfully discriminating GTHCs, NTHCs, and NHCs.

**Table 1:** GTHC biomarker candidates in mouse liver proposed by Kossler et al. [46]

Up-regulated genes by GTHC:	
DNA damage response:	Bax, Bcl2a1, Ccng1, Ddit4l, Emp3, Enc1, Iqgap1, Map3k20, Mgmt, Phlda3, Pierce1, Siva1, Top2a, Tspan13, Zeb2
Cellular assembly and organization:	Col1a2, Fbn1, Fstl1, Loxl2, Nisch, Plekha2, Tagln2, Tmsb10, Tuba1a
Immune response:	Ccr2, Cd34, Fgl2, H2-Dma, H2-DMb2, Lck, Mbl2
Detoxification response:	Ces2e, Gstp3
Others:	Acot9, Akap13, Atp6v1d, Ccdc80, Cox6b2, Exoc4, G6pdx, Ggta1, Pqlc3, Snx6, Zdhhc14, Zfp54, Zfp958
Down-regulated genes by GTHC:	
DNA damage response:	Bcor
Others:	Dleu2, Ltn1, Moxd1, Srprb

**Table 2:** NGTHC biomarker candidates in mouse liver proposed by Kossler et al. [46]

Up-regulated genes by NGTHC:	
Cell cycle progression:	Hnf4aos (0610008F07Rik), Nsl1, Rorc
Apoptosis:	Pgap2
Detoxicification response:	Ces2a, Cyp2c250, Cyp2c65, Gstm1
Cellular assembly and organization:	Nebl
Others:	Akr1d1, Atosa, Atxn10, Dgka, Fam171a1, Fndc5,
	Ginm1 (BC013529Rik), Gm10419, Gm2011, Pnliprp1,
	Tulp2, Zkscan14, 2810433D01Rik, 4930597L12Rik, 4931406C07Rik
Down-regulated genes by NGTHC:	
DNA damage response:	Armt1
Cell cycle progression:	Aigl, Atad2, Fgl1, Mcm5, Ncapg2, Pola1, Prkd2, Tead1
Apoptosis:	Nolc1, Tnfrsf1b
Cellular assembly and organization:	Pkp2
Immune response:	Cebpb
Others:	Camkk2, Coa6 (1810063B05Rik), Gnat1, Grhl1, Grk3, Gtf2b, Hip1r,
	Nr2c2, Pla2g16, Prdm15, Rasal2, Samd4a, Slc25a32, Tmem98,
	Tmem181c-ps, Tmem268, Zfp472, Zfp750, A930036K24Rik,
	2310075K07Rik, 5430416B10Rik,

### Discrimination of GTHCs and NGTHCs and/or NGTNHCs in public DNA microarray data by PCA

**Fig. 4:**
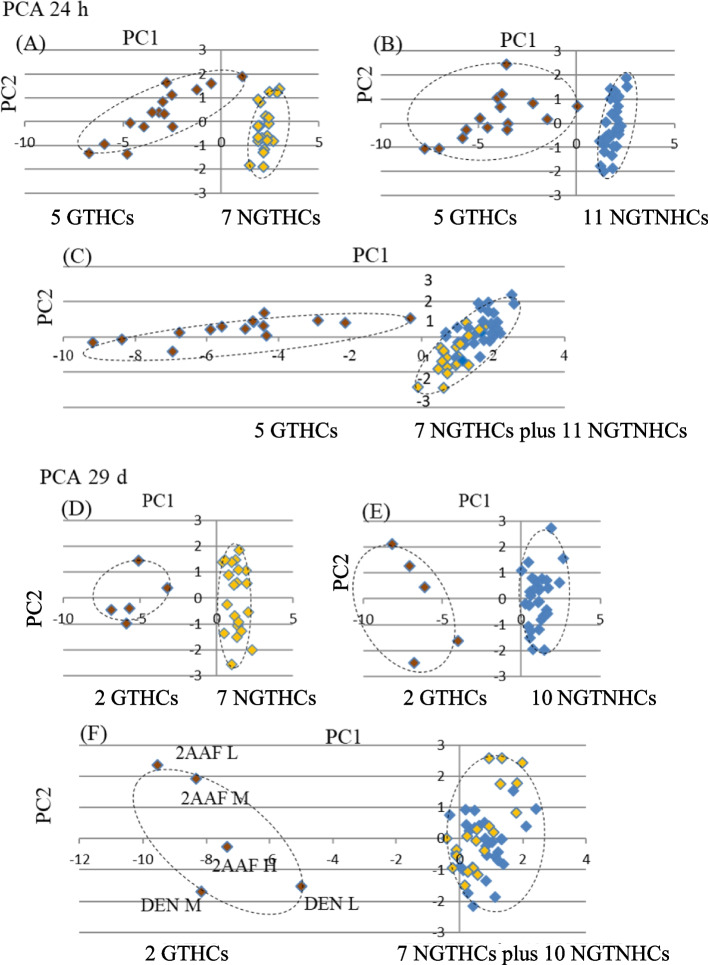
Analysis of rat liver public data (OPEN TG-GATEs, DNA microarray). Discrimination of GTHCs from NGTHCs and NGTNHCs at 24 h after a single administration and 29 days after repeated administrations by PCA with 12 marker genes (*Aen*, *Bax*, *Btg2*, *Ccnf*, *Ccng1*, *Cdkn1a*, *Gdf15*, *Lrp1*, *Mbd1*, *Phlda3*, *Plk2,* and *Tubb2c*). The mean of each control group was calculated as 0 (log2), and ratio (exp/cont) log2 was calculated. These numerical values were analyzed by PCA. At 24 h, five GTHCs (brown-colored, AAF, AFL, DEN, NNM, and 2NF) were discriminated from seven NGTHCs (yellow-colored, CLO, ETH, FEN, GEM, HEX, PHE, and WY) (**A**); and five GTHCs (AAF, AFL, DEN, NNM, and 2NF) were discriminated from 11 NGTNHCs (blue-colored, AA, ASP, CAF, CPA, CPP, DEX, DIA, IND, PBZ, THE, and TOL) (**B**), with each of the three doses (low, middle and high) and five GTHCs to seven NGTHCs plus 11 NGTNHCs (**C**). At 29 days, two GTHCs (AAF and DEN) were discriminated from seven NGTHCs (CLO, ETH, FEN, GEM, HEX, PHE, and WY) (**D**), two GTHCs (AAF and DEN) from 10 NGTNHC (AA, ASP, CAF, CPA, CPP, DIA, IND, PBZ, THE, and TOL) (**E**), and two GTHCs from seven NGTHCs plus 10 NGTNHCs (**F**), with each of the three doses (low, middle and high except DEN). Each group is discriminated with a dashed line. GTHCs [AAF: 2- acetamidofluorene, AFL: aflatoxin B1, 2NF: 2-nitrofluorene, DEN: *N*-nitrosodiethylamine and NNM: N-nitrosomorpholine], NGTHCs [CLO: clofibrate, ETH: ethanol, FEN: fenofibrate, GEM: gemfibrozil, HEX: hexa-chlorobenzene, PHE: phenobarbital, and WY: WY-14643] and NGTNHCs (mostly pharmaceutical drugs) [AA: allyl alcohol, ASP: aspirin, CAF: caffeine, CPA: chlorpheniramine, CPP: chlorpropamide, DEX: dexamethasone, DIA: diazepam, IND: indomethacin, PBZ: phenylbutazone, THE: theophylline, and TOL: tolbutamide]. Each group is enclosed with a dashed ellipse [47]

Furihata and Suzuki analyzed in vivo rat data from the public DNA microarray data, in Open TG-GATEs [(Database Description—Open TG-GATEs | LSDB Archive (biosciencedbc.jp)] with the 12 mouse marker genes (*Aen*, *Bax*, *Btg2*, *Ccnf*, *Ccng1*, *Cdkn1a*, *Gdf15*, *Lrp1*, *Mbd1*, *Phlda3, Plk2*, and *Tubb4b*) (JEMS/MMS marker genes) [47]. They analyzed the data associated with exposure to a total of 23 chemicals: 5 typical rat GTHCs (2AAF, AFB1, 2-nitrofluorene, DEN, and N-nitrosomorpholine), 7 typical rat NGTHCs (clofibrate, ethanol, fenofibrate, gemfibrozil, hexachlorobenzene, phenobarbital, and WY-14643), and also 11 NGTNHCs (allyl alcohol, aspirin, caffeine, chlorpheniramine, chlorpropamide, dexamethasone, diazepam, indomethacin, phenylbutazone, theophylline, and tolbutamide) from Open TG-GATEs. The analysis was performed 3, 6, 9, and 24 h after a single administration and 4, 8, 15, and 29 days after repeated administrations. Genes that were differentially expressed in a dose-dependent manner that was specific to GTHCs were observed, and their significance was assessed using the Williams test during 3–24 h and 4–29 days. PCA successfully discriminated GTHCs from NGTHCs and NGTNHCs at 24 h and 29 days, as shown in Fig. 4 [47]. The results demonstrated that 12 previously proposed mouse marker genes (JEMS/MMS marker genes) are useful for discriminating rat GTHCs from NGTHCs and NGTNHCs.

In another study, Kanki et al. studied 13 NGTHCs with various MOA from OPEN TG-GATEs (28 days) and selected 42 genes that were up-regulated and 8 that were down-regulated upon exposure to them [48]. However, none of them coincided with the 55 genes associated with NGTHCs exposure proposed by Kossler et al. [46]. It is considered that the reason for this discrepancy is that NGTHCs were compared only with the control but not with GTHCs in the study [48].

### Discrimination of GTHCs from NGTHCs using RNA-Seq in short-term in vivo test

**Fig. 5:**
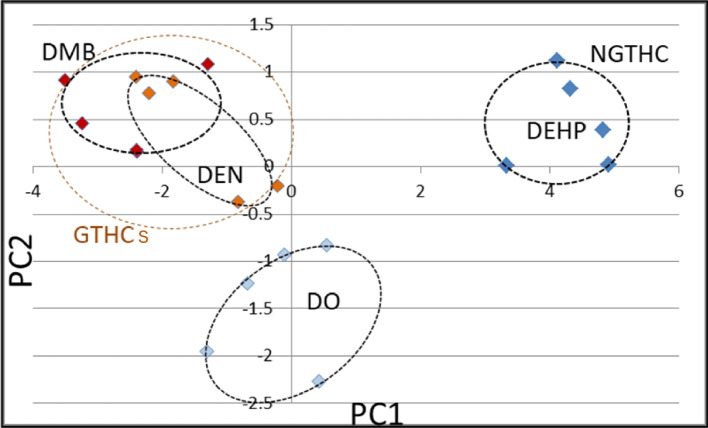
Analysis by RNA-Seq in rat liver after 28 days of repeated treatment. Discrimination of typical GTHCs (DEN and DMB) to a typical NGTHC (DEHP) and DO by PCA. The mean of each control group was calculated as 0 (log2) and ratio (exp/cont) log2 was calculated. GTHCs (DEN, orange and DMB, brown) were differentiated from DEHP (blue) with PC1. DO (pale blue) was differentiated from typical GTHCs (DEN and DMB) and a typical NGTHC (DEHP). DEN: *N*-nitrosodiethylamine, DMB: 3,3-dimethylbenzidine·2HCl, DEHP: di(2-ethylhexyl)phthalate, and DO: 1,4-dioxane [8]

**Fig. 6:**
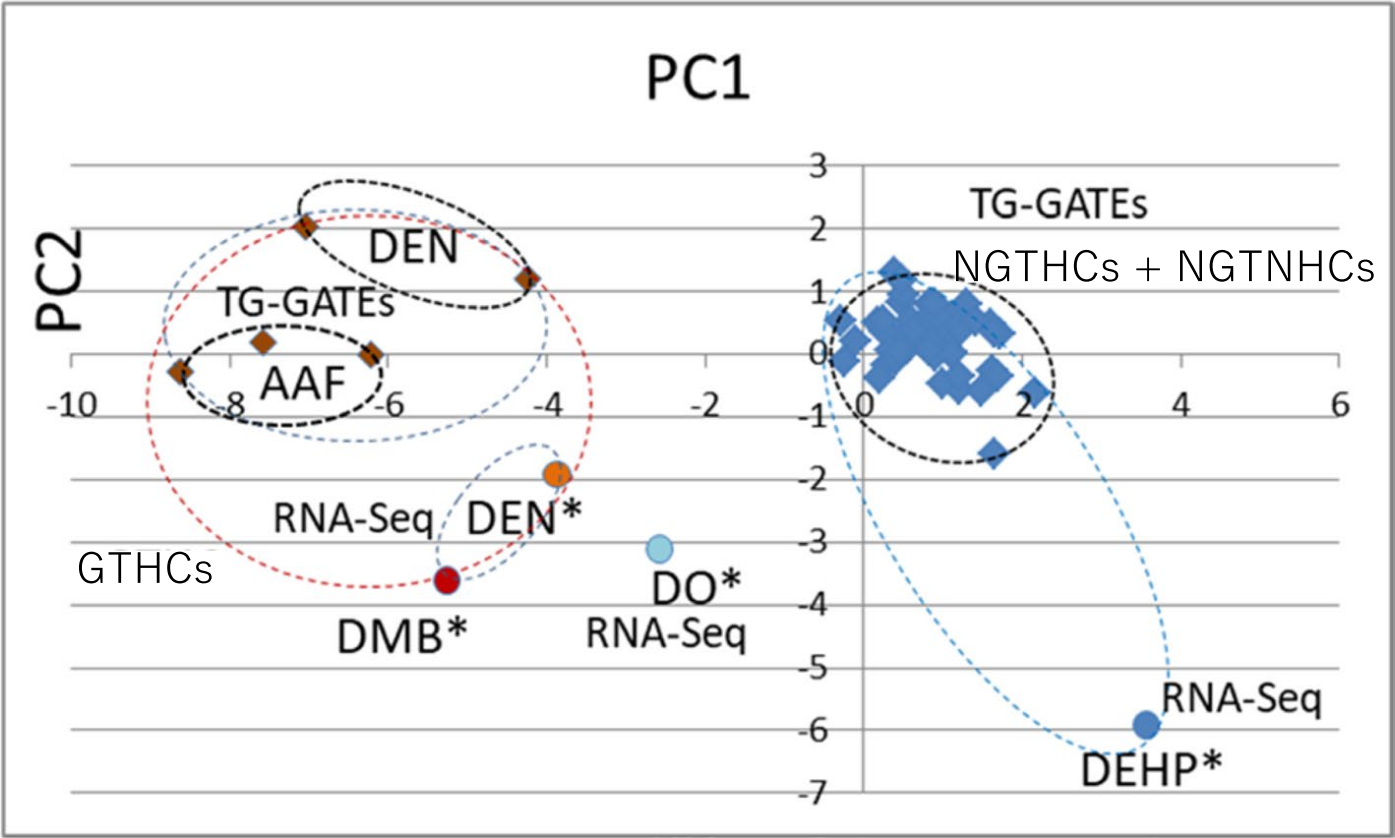
PCA analysis of the results of RNA-Seq experiment together with our previous analysis of public data from TG-GATEs [47]. DEN* (dark orange), DMB* (brown), DEHP* (blue), and DO* (pale blue) are from the RNA-Seq experiment. Four typical GTHCs [DEN* (RNA-Seq, dark orange), DMB* (RNA-Seq, light brown), DEN (TG-GATEs, dark brown), and AAF (GT-GATEs, dark brown)] were clearly discriminated from eight NGTHCs [DEHP* (RAN-Seq, blue) and 7 NGTHCs [(TG-GATEs, blue), clofibrate, ethanol, fenofibrate, gemfibrozil, hexachlorobenzene, phenobarbital, and WY-14613] plus 10 NGTNHCs [(TG-GATEs, blue), allyl alcohol, aspirin, caffeine, chlorpheniramine, chlorpropamide, diazepam, indomethacin, phenylbutazone, theophylline, and tolbutamide] with PC1. However, DO* (pale blue) from RNA-Seq data may be intermediate between typical GTHCs and the group of typical NGTHCs plus NGTNHCs. Each point shows the mean of five animals for RNA-Seq [8] and three animals for TG-GATEs [47]

Furihata et al. used intact RNA derived from freshly frozen rat liver tissues after 4 weeks of the feeding of chemicals in the water or the food [8]. Using targeted RNA-Seq with specific primers for 12 candidate marker genes (JEMS/MMS marker genes) previously proposed by Furihata and Suzuki [47] and sample-specific sequence tags, they evaluated the rat hepatocarcinogen 1,4-dioxane (DO) with ambiguous genotoxicity compared with typical GTHCs, DEN and 3,3-dimethylbenzidine·2HCl (DMB), and an NGTHC, DEHP. Gene expression profiles of the 12 genes under DO treatment differed significantly from those with DEN and DMB, as well as DEHP. Finally, PCA successfully differentiated GTHCs from DEHP and DO using these 11 genes (*Aen*, *Bax*, *Btg2*, *Ccnf*, *Ccng1*, *Cdkn1a*, *Lrp1*, *Mbd1*, *Phlda3, Plk2*, and *Tubb4b*), as shown in Figs. 5 and 6 [8]. The present results suggest that RNA-Seq and PCA are useful for differentiating typical GTHCs and typical NGTHCs in the rat.

### Discrimination of a GTHC from an NGTHC using RNA-Seq with formalin-fixed paraffin-embedded (FFPE) samples

**Fig. 7:**
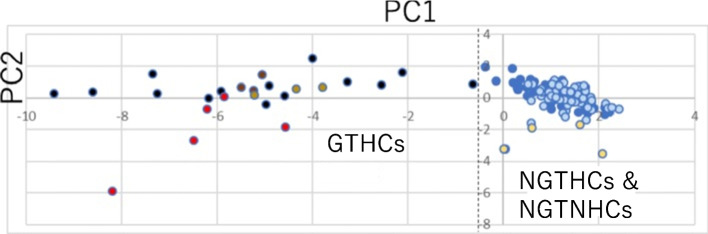
Discrimination of FFPE-AAF from FFPE-CRE [9] together with the previous rat GTHCs, NGTHCs, and NGTNHCs calculated from public Open TG-GATEs data [47] using PCA. FFPE data show individual results and TG-GATEs data show the mean of three rats at each point. Red: FFPE-AAF, brown: AAF at 24 h from Open TG-GATEs, light brown: AAF on 29 days from Open TG-GATEs, black: GTHCs from Open TG-GATEs. Yellow: FFPE-CRE, blue: NGTHCs from Open TG-GATEs, light blue: NGTNHCs from Open TG-GATEs. Two points of FFPE-CRE (− 0.042/ − 3.26 and − 0.08/ − 3.26) overlapped. Five typical GTHCs [2-acetamidofluorene (AAF), AFL, DEN, 2NF, and NNM at 24 h and AAF and DEN on 29 daysin Open TG-GATEs data] were separated from the seven typical NGTHCs (CLO, ETH, FEN, GEM, HEX, PHE, and WY at 24 h and 29 days in TG-GATEs data) and eleven NGTNHCs (AA, ASP, CAF, CPA, CPP, DEX, DIA, IND, PBZ, THE, and TOL at 24 h and 29 days in Open TG-GATEs data) using PCA. Two groups of GTHCs and (NGTHCs and NGTNHCs) were separated using PC1 (− 0.637 for DEN 24L against − 0.159 for FEN 24 M). The dashed line is the border line of the two groups. FFPE-AAF in the GTHCs group was separated from FFPE-CRE grouped in NGTHCs [9]

Furihata et al. used RNA-Seq with FFPE samples from rat liver tissues after 4 weeks of the feeding of chemicals in the water or the food [9]. Specifically, targeted RNA-Seq was applied to FFPE samples to analyze 12 genes (JEMS/MMS marker genes) as potential markers for rat responses to GTHCs and NGTHCs, with the comparison between a typical GTHC, 2AAF, and p-cresidine (CRE), the genotoxicity of which is ambiguous. 2AAF induced remarkable differences in the expression of eight genes (*Aen*, *Bax*, *Btg2*, *Ccng1*, *Gdf15*, *Mbd1*, *Phlda3*, and *Tubb4b*) from that in the control group, while CRE only induced expression changes in *Gdf15*, as shown by Tukey’s test. Meanwhile, gene expression profiles for nine genes (*Aen*, *Bax*, *Btg2*, *Ccng1*, *Cdkn1a*, *Gdf15*, *Mbd1*, *Phlda3*, and *Plk2*) differed between samples treated with 2AAF and CRE. Finally, PCA of 12 genes (*Aen*, *Bax*, *Btg2*, *Ccnf*, *Ccng1*, *Cdkn1a*, *Gdf15*, *Lrp1*, *Mbd1*, *Phlda3*, *Plk2*, and *Tubb4b*) (JEMS·MMS marker genes) using our previous Open TG-GATE data [47] plus 2AAF and CRE successfully differentiated 2AAF, as a GTHC, from CRE, as an NGHTC (Fig. 7) [9]. It was thus concluded that targeted RNA-Seq on FFPE samples and PCA are useful for evaluating a typical rat GTHC and an NGTHC.

### Recent whole-transcriptome RNA-Seq reports on in vivo analyses in animal liver, kidney, and other organs

#### Liver

Various whole-transcriptome RNA-Seq studies on the effects of hepatocarcinogens in rodent liver have been reported [5, 6, 10–18, 23], although they did not examine the discrimination of GTHCs from NGTHCs.

Li et al. examined rat livers treated with a GTHC, AFB1, for 5 days and analyzed the effects using RNA-Seq, TempO-Seq, DNA microarray, and qPCR. They showed that RNA-Seq revealed toxicological insights from pathway enrichment, with overall higher statistical power compared with TempO-seq and DNA microarray. They detected 862 DEGs (491 up-regulated and 371 down-regulated by AFB1) in HiSeq2000 and confirmed 11 up-regulated genes (*Ccnb1*, *Cenpw*, *G6pd*, *Nt5dc2*, *Pttg1*, *Spp1*, *Stmn1*, *Tacc3*, *Tk1*, *Ube2c*, and *Ube2t*) by qPCR [10].

In another study, Nault et al*.* examined an NGTHC, acetamide, in rat liver after treatment for 7 and 28 days. They showed the DEGs results using heatmaps. They reported 9 up-regulated genes: (*E2f4*, *Ar*, *Mybl1*, *Kdm6a*, *Sox2*, *Mycn*, *Sry*, *Mybl2*, and *EF1*) and 10 down-regulated ones: (*Esr1*, *Rxr, Ppara*, *LXRalpha*, *Pparg*, *Cebpa*, *Egr1*, *Cebpb*, *Foxo1*, and *Foxp1*). Additionally, they wrote complex increase/decrease in the following genes *Hebp2*, *Acot1*, *Ifit1*, *Cenpw*, *Chek2*, *Parpbp*, *Cyp17a1*, *Slc7a1*, and *Prom1* in the paper [11].

Elsewhere, Gong et al*.* reported that the US FDA-led SEQC (i.e., MAQC-III) project conducted a comprehensive study focusing on the transcriptome profiling of rat liver samples treated with 27 chemicals with various MOA for 3 to 7 days to evaluate the utility of RNA-Seq in safety assessment and elucidating the mechanism of toxicity [12].

Moreover, Bushel et al. examined the effects of treatment with 15 chemicals with various MOA for 3 to 7 days in rat liver and presented the data obtained by DNA microarray, RNA-Seq, and Tempo-Seq in a heatmap [13].

#### Kidney

Li et al. studied the effects of a carcinogenic dose of aristolochic acid for 12 weeks in rat kidney.

Four thousand fifty one up-regulated and 2743 down-regulated mRNAs were observed and 43 up-regulated and 20 down-regulated miRNAs were observed as measured by PCA and hierarchical clustering analysis [19].

#### Lung

Israel et al. reported DEGs induced by a GTC, 1,3-butadiene, in mouse liver, lung, and kidney for 2 weeks. They performed RNA-Seq, identification of accessible chromatin (ATAC-seq), and characterization of regions with histone modifications associated with active transcription (ChIP-seq for acetylation at histone 3 lysine 27, H3K27ac). Most changes were restricted to lung tissue. The results were shown in heatmaps. They showed that the DEGs were involved in Phase I metabolism (58 Cyp family members and 12 others), Phase II metabolism (58 genes), and IFNγ signaling (75 genes) [15].

Additionally, Felley-Bosco and Rehrauer reported RNA-Seq data from asbestos-exposed mice. In that study, an asbestos suspension was injected every 3 weeks for eight rounds and an examination was performed 33 weeks after the first injection. They performed data mining of publicly available datasets to evaluate how noncoding RNA contributes to mesothelioma heterogeneity. Nine noncoding RNAs (*Fendrr*, *Gm26902*, *Gm17501*, *Meg3*, *miR 17–92 cluster*, *Dubr*, and *Firre*) were specifically elevated in mesothelioma tumors and shown to contribute to the heterogeneity of human mesothelioma. Because some of these RNAs have known oncogenic properties, this study supported the concept that noncoding RNAs can act as cancer progenitor genes [20].

#### Colon

Guo et al. reported mechanisms of mouse colitis-accelerated colon carcinogenesis induced by azoxymethane/dextran sulfate sodium treatment for 22 weeks. The 10 most up-regulated genes in tumors were *Alb*, *Alox15*, *Clca4*, *Cxcl6*, *Lyz*, *Mmp7*, *Mmp10*, *Pnliprp 1*, *Slc30a2*, and *Wif1*, while the 10 most down-regulated ones were *Ca3*, *Chrna*3, *Folh1, Nos1*, *Pln*, *Retnlb*, *Sst*, *Stmn3, Sycn*, and *Zcchc12* [21].

#### Pancreas

Asahina et al*.* reported that alcohol intake for 5 months induced pancreatic ductal adenocarcinoma in *Pdx1* ^Cre^; LSL-*Kras* ^G12D^ mutant mice. Whole RNA-seq analysis revealed that the consumption of alcohol increased the expression of markers for tumors (*Epcam*, *Krt19*, *Prom1*, *Wt1*, and *Wwtr1*), stroma (*Dcn*, *Fn1*, and *Tn*c), and cytokines (*Tgfb1* and *Tnf*) and decreased the expression of *Fgf21* and *Il6* in the pancreatic tumor tissues [22].

### Discussion

Kinaret et al. [49] asserted that, although the advent of high-throughput hybridization-based technologies, such as DNA microarrays, significantly boosted the generation of large-scale gene expression profiles, recent advances in sequencing technologies further improved such capability. For instance, RNA-Seq allows the detection of gene expression with an increased dynamic range, solving the problem of probe saturation for highly expressed transcripts. Furthermore, RNA-Seq does not need a priori knowledge about the genomic sequence of the studied organism and does not suffer from the above-mentioned cross-hybridization events, especially in the analysis of complex genomes. As a consequence, RNA-Seq allows de novo transcript discovery to be performed to identify unannotated transcripts and characterize new transcripts generated by alternative splicing. However, an appropriate analytical plan should be made to avoid or mitigate certain biases that could occur during the data management and analysis. For instance, previous works [49] showed that standard normalization procedures can affect the sensitivity of differential expression analysis, reflecting the behavior of a relatively small number of either high-count or ubiquitous genes. RNA-Seq typically produces larger and more complex data, which require more time and more sophisticated analytical approaches, than in DNA microarray experiments, for example. Although transcriptome profiling is increasingly being employed in toxicogenomic experiments, the analytical pipelines are still far from being standardized. To date, no benchmark of the optimal analytical procedures in transcriptome profiling in toxicogenomic experiments has been formulated. Recently, the reduction of the cost of analyzing a single transcriptome made the accomplishment of large-scale studies possible, which have been carried out by international programs such as CMAP, TOX21, and LINCS1000 [49].

Comparing RNA-Seq with qPCR and DNA microarray, RNA-Seq is reflecting the absolute amount of RNA expression more directly than others as read counts. The reliability of the results can be confirmed by sequence without a disturbance of mismatch in probes or primers and is applicable for alternative splicing. The qPCR method is easy to perform and does not require advanced experience but is applicable only after the selection of target genes. It is not a comprehensive method compared to total RNA-seq or DNA microarray. The DNA microarray methods require many steps and skills and have more variances among different platforms. The reliability of the results is slightly lower than the other two methods. The major results should often be confirmed by qPCR. From the analysis of previous DNA microarray papers, we have learned that the marker genes differ depending on the type of chemicals studied. The marker genes in previous DNA microarray papers do not always match. It would be useful to examine published DNA microarray papers to identify candidate marker genes, and it would be useful to accumulate RNA-Seq (whole) data, which is more reliable than DNA microarray, to converge the marker genes. This requires easy-to-use bioinformatics.

Kinaret et al. [49] introduced the following public data.

- Chemical Effects in Biological Systems (CEBS, Chemical Effects in Biological Systems; nih.gov) [50, 51],
- Connectivity Map (CMAP, Connectivity Map, Broad Institute) [52],
- LINCS 1000 NIH LINCS Program (lincsproject.org) [53],
- DrugMatrix (norecopa.no) [54],
- Open TG-GATEs (LSDB Archive; biosciencedbc.jp) [55],
- ArrayExpress (EMBL-EBI) [56], and
- Gene Expression Omnibus (GEO; nih.gov) [57, 58].

The qPCR has been used as an efficient screening method after narrowing down biomarker genes by comprehensive analysis using DNA microarray. Similarly, “targeted” RNA-Seq, in which specific PCR primers are designed to amplify only selected gene transcripts, can be used. In “targeted” RNA-Seq, the unique sequencing tag allows a large number of samples to be mixed and sequenced at the same time, making it a simpler and more cost-effective method than qPCR. To increase the efficiency of the analysis, it is recommended to combine genes with similar expression levels for “targeted” RNA-Seq [8].

The next newly established technology for RNA-Seq is single-cell RNA-Seq (scRNA-Seq). The scRNA-Seq pipeline has emerged as a valuable tool for uncovering individual cellular functions in thousands to millions of cells, an advancement over the bulk RNA-seq method of averaging gene expression across all cells in a tissue [59]. However, to the best of our knowledge, scRNA-Seq has yet to be applied to toxicogenomics, including to the discrimination of GTCs, NGTCs, and NCs.

When discussing the proposed candidate genes that can act as markers of GTHCs and NGTHCs in RNA-Seq, DNA microarray, and qPCR data on samples from rodent liver, they are not always consistent among different published papers [5–31, 36–47]. For example, JEMS·MMS·Toxicogenomics group proposed 12 candidate genes (*Aen*, *Bax*, *Btg2*, *Ccnf*, *Ccng1*, *Cdkn1a*, *Gdf15*, *Lrp1*, *Mbd1*, *Phlda3*, *Plk2*, and *Tubb4b*) to discriminate GTHCs from NGTHCs and NGTNHCs by PCA from analyses of mouse liver [41], rat liver [43], public DNA microarray data (OPEN TG-GATEs) [47], RNA-Seq [8], and RNA-Seq on FFPE samples [9] upon 4 h to 28 days of treatment with a total of 35 chemicals (15 GTHCs, 9 NGTHCs, and 11 NGTNHCs). Meanwhile, Kossler et al*.* proposed 51 marker candidate genes that could differentiate GTHCs from NGTHCs and NHCs (Table 1) and 58 marker candidate genes that could differentiate NGTHCs from GTHCs and NHCs (Table 2) in mouse liver examined by DNA microarray. They examined a total of 13 chemicals (3 GTHCs, 6 NGTHCs, and 4 NHCs) in mouse liver after treatment for 3 and 14 days [46]. They proposed 15 genes involved in the DNA damage response, four of which (Bax, *Ccng1*, *Ddit4l*, and *Phlda3*) overlapped with those in the studies of JEMS·MMS·Toxicogenomics group. However, Kossler et al*.* examined three GTHCs that differed from the 15 GTHCs examined by JEMS·MMS·Toxicogenomics group. Moreover, Park et al. presented significantly deregulated genes in rasH2 mouse liver upon treatment with DEN and 2AAF; there were 47 upregulated genes, including *Aen*, *Bax*, *Btg2*, *Ccng1*, *Cdkn1a*, *Ddit4l*, *Plk2*, *Mdm2*, *Phlda3*, and *Tubb4b*, which were also proposed by JEMS·MMS·Toxicogenomics group, and 11 downregulated genes [47]. JEMS·MMS·Toxicogenomics group also studied DEN and 2AAF. Furthermore, Jonker et al. reported the discrimination of 2 GTCs, 2NGTCs and 2 NGTNCs in the liver of both wild-type and DNA repair-deficient Xpa2/2/p531/2 (Xpa/p53) mice using DNA microarray and heatmap [60]. However, their candidate genes differed from those in other published papers. Finally, Li et al. examined rat liver upon treatment with a GTHC, AFB1, for 5 days and performed analyses using RNA-Seq, TempO-Seq, DNA microarray, and qPCR. They proposed 11 completely different marker genes in other published papers [10]. Given these conflicting findings, it should be useful to reselect or validate genes from all available databases to discriminate GTCs, NGTCs, and NGTNCs.

In connection with restrictions on animal testing, “OECD Guidelines for the Testing of Chemicals, [Repeated Dose 28-Day Oral Toxicity Study in Rodents (OECD TG 407)] [Test No. 407: Repeated Dose 28-day Oral Toxicity Study in Rodents | READ online (oecd-ilibrary.org)] is still valid for testing chemical toxicity. This assay determines the general toxicity of chemicals in rodents after 28 days of oral dosing (e.g., effects on the liver, kidneys, heart, and lungs). Despite restrictions being placed on animal testing, this test will continue to be applied. We can use the animal organs from the test collaboratively and use the samples, which would reduce the number of experimental animals used.

In toxicogenomic experiments, there are protocol issues to be considered, such as the method and number of administered doses, dose setting, and timing of observation. As yet, no consensus has been reached on the optimal settings for these variables. Therefore, it would be beneficial to adjust the strategy according to each study to find the best protocol, but also to adjust settings to match previous studies, such as using a 28-day repeated dosing test in rats. Regarding the future direction of toxicogenomics concerning the 3Rs concept, we also propose incorporating not only toxicogenomics but also other genotoxicity assays (e.g., micronucleus test, error-corrected sequencing, comet assay, DNA adduct analysis) into 28-day repeated dosing study in rats to enable a reduction in the number of animals used by applying multi-endpoint assays.

Targeted RNA-Seq requires only a few hundred base pairs for sequencing, which enables the use of RNA from FFPE samples. A large number of FFPE samples from pathological examinations in previous studies are available, including those from 2-year rodent bioassays for carcinogenicity. The examination of stored FFPE samples would enable the establishment of substantial expression data with information on toxicological endpoints such as carcinogenicity [61]. The construction of a large database with data on a large set of genotoxic carcinogens would improve the efficiency and reliability of biomarker genes for discriminating such compounds.

### Conclusions

There is a growing need to develop alternative in vivo methods to the 2-year rodent bioassay to assess the carcinogenicity of environmental chemicals. Toxicogenomics, including recent RNA-Seq and previous qPCR and DNA microarray, has been studied for its potential as a short-term in vivo alternative to long-term animal studies. RNA-Seq has identified more DEGs and provided a wider quantitative range of expression level changes than conventional DNA microarrays. JEMS·MMS·Toxicogenomics group successfully discriminated GTHCs from NGTHCs and/or NGTNHCs in rat and mouse liver by 12 marker genes using targeted RNA-Seq, RNA-Seq on FFPE samples, qPCR, and DNA microarray with PCA as a statistical approach. The 12 marker genes were re-validated by public DNA microarray data (OPEN TG-GATEs). EPA studied DEGs induced by 4 known GTHCs in rat liver using DNA microarray and proposed 7 biomarker genes, four of which (*Bax*, *Btg2*, *Ccng1*, *and Cdkn1a*) overlapped with those of JEMS/MMS 12 genes. Candidate genes published using RNA-Seq, qPCR, and DNA microarray will be useful for the future development of short-term in vivo studies of environmental carcinogens using RNA-Seq. In connection with the restrictions on animal testing and the 3Rs concept, it would be beneficial to adjust settings to match a 28-day repeated dosing test in rats rather than seeking the best protocol for toxicogenomics.

## Supplementary Information

**Additional file 1.** Short-term in vivo tests for carcinogens by RNA-Seq, DNA microarray, and qPCR.

## Data Availability

Not applicable.

## Words

DEGs: Differentially expressed protein-coding genes
EFPIA: European Federation of Pharmaceutical Industries and Associations
FFPE: Formalin-fixed paraffin-embedded
GTC: Genotoxic carcinogen
GTHC: Genotoxic hepatocarcinogen
IMI: Innovative Medicines Initiative
JEMS·MMS: The Japanese Environmental Mutagen Society ·Mammalian Mutagenicity Study Group
MARCAR: Biomarkers and molecular tumor classification for non-genotoxic carcinogenesis
MOA: Mode of action
NC: Non-carcinogen
NGTC: Non-genotoxic carcinogen
NGTHC: Non-genotoxic hepatocarcinogen
NGTNHC: Non-genotoxic non-hepatocarcinogen
NHC: Non-hepatocarcinogen
OECD: Organization for Economic Co-operation and Development
qPCR: Quantitative real-time PCR
PCA: Principal component analysis
RNA-Seq: Next-generation RNA sequencing
SVM: Support vector machine
TempO-Seq: Templated Oligo-Sequencing

## Chemicals

AAF, 2AAF: 2-Acetylaminofluorene
AFB1: Aflatoxin B1
CRE: *p*-Cresidine
DEHP: Di(2-ethylhexyl)phthalate
DEN: Diethylnitrosamine
DES: Diethylstilbestrol
DMN: Dimethylnitrosamine
DO: 1,4-Dioxane
ENU: Ethylnitrosourea
Mpy: Methapyrilene
2-NF: 2-Nitrofluorene
NNK: 4-(Methylnitrosamino)-1-(3-pyridyl)-1-butanone
PBO: Piperonylbutoxide
WY: Wy-14643

## Genes

*Acot1*: Acyl-CoA thioesterase 1
*Acot9*: Acyl-CoA thioesterase 9
*Aen*: Apoptosis enhancing nuclease
*Akap13*: A-kinase anchoring protein 13
*Akr1d1*: Aldo-keto reductase family 1 member D1
*Alb*: Albumin
*Alox15*: Arachidonate 15-lipoxygenase
*Apex1*: Apurinic/apyrimidinic endodeoxyribonuclease 1
*Ar*: Androgen receptor
*Armt1*: Acidic residue methyltransferase 1
*Atad2*: ATPase family AAA domain containing 2
*Atosa*: Atos homolog A
*Atp6v1d*: ATPase, H + transporting lysosomal V1 subunit D
*Atxn10*: Ataxin 10
*Bax*: BCL2 associated X, apoptosis regulator
*Bcl2a1*: BCL2 related protein A1
*Bcor*: BCL6 interacting corepressor
*Btg2*: BTG anti-proliferation factor 2
*Ca3*: Carbonic anhydrase 3
*Camkk2*: Calcium/calmodulin-dependent protein kinase kinase 2
*Ccdc80*: Coiled-coil domain containing 80
*Ccnb1*: Cyclin B1
*Ccnf*: Cyclin F
*Ccng1*: Cyclin G1
*Ccr2*: C-C motif chemokine receptor 2
*Cd34*: CD34 antigen
*Cdk1*: Cyclin-dependent kinase 1
*Cdkn1a*: Cyclin-dependent kinase inhibitor 1A
*Cebpa*: CCAAT/enhancer binding protein alpha
*Cebpb*: CCAAT/enhancer binding protein beta
*Chek2*: Checkpoint kinase 2
*Cenpw*: Centromere protein W
*Ces2a*: Carboxylesterase 2A
*Ces2e*: Carboxylesterase 2E
*Chrna*3: Cholinergic receptor, nicotinic, alpha polypeptide 3
*Clca4*: Chloride channel accessory 4
*Coa6*: Cytochrome c oxidase assembly factor 6
*Col1a2*: Collagen, type I, alpha 2
*Cox6b2*: Cytochrome c oxidase subunit 6B2
*Cxcl6*: C-X-C motif chemokine ligand 6
*Cyp2c65*: Cytochrome P450, family 2, subfamily c, polypeptide 65
*Cyp17a1*: Cytochrome P450, family 17, subfamily a, polypeptide 1
*Dcn*: Decorin
*Ddit4l*: DNA-damage-inducible transcript 4-like
*Dgka*: Diacylglycerol kinase, alpha
*Dleu2*: Deleted in lymphocytic leukemia, 2
*Dubr*: Dppa2 upstream binding RNA
*E2f4*: E2F transcription factor 4
*EF1*: Elongation factor 1-alpha
*Egr1*: Early growth response 1
*Emp3*: Epithelial membrane protein 3
*Enc1*: Ectodermal-neural cortex 1
*Epcam*: Epithelial cell adhesion molecule
*Esr1*: Estrogen receptor 1
*Exoc4*: Exocyst complex component 4
*Fam171a1*: Family with sequence similarity 171, member A1
*Fbn1*: Fibrillin 1
*Fendrr*: Foxf1 adjacent non-coding developmental regulatory RNA
*Fgf21*: Fibroblast growth factor 21
*Fgl1*: Fibrinogen-like protein 1
*Fgl2*: Fibrinogen-like protein 2
*Firre*: Functional intergenic repeating RNA element
*Fn1*: Fibronectin 1
Fndc5: Fibronectin type III domain containing 5
*Folh1*: Folate hydrolase 1
*Foxo1*: Forkhead box O1
*Foxp1*: Forkhead box P1
*Fstl1*: Follistatin-like 1
*G6pd*: Glucose-6-phosphate dehydrogenase
*G6pdx*: Glucose-6-phosphate dehydrogenase X-linked
*Gapdh*: Glyceraldehyde-3-phosphate dehydrogenase
*Gdf15*: Growth differentiation factor 15
*Ggta1*: Glycoprotein alpha-galactosyltransferase 1
*Ginm1*: Glycoprotein integral membrane 1
*Gm2011*: Predicted gene 2011
*Gm10419*: Predicted gene 10419
*Gm17501*: Predicted gene, 17,501
Gm26902: PRKCA-binding protein
Gnat1: G protein subunit alpha transducin 1
*Grhl1*: Grainyhead like transcription factor 1
*Grk3*: G protein-coupled receptor kinase 3
*Gstm1*: Glutathione S-transferase, mu 1
*Gstp3*: Glutathione S-transferase pi 3
*Gtf2b*: General transcription factor IIB
*H2-Dma*: Histocompatibility 2, class II, locus Dma
*H2-DMb2*: Histocompatibility 2, class II, locus Mb2
*Hebp2*: Heme binding protein 2
*Hip1r*: Huntingtin interacting protein 1 related
*Hnf4aos*: Hepatic nuclear factor 4 alpha, opposite strand
*Hspd1*: Heat shock protein family D (Hsp60) member 1
*Hspa9*: Heat shock protein family A (Hsp70) member 9
*Ifit1*: Interferon-induced protein with tetratricopeptide repeats 1
*Il6*: Interleukin 6
*Iqgap1*: IQ motif containing GTPase activating protein 1
*Kdm6a*: Lysine demethylase 6A
*Krt19*: Keratin 19
*Loxl2*: Lysyl oxidase-like 2
*Lck*: Lymphocyte protein tyrosine kinase
*Lrp1*: LDL receptor related protein 1
*Ltn1*: Listerin E3 ubiquitin protein ligase 1
*lxr*: LexA regulated function (*Escherichia phage P1)*
*Lyz1*: Lysozyme 1
*Map3k20*: mitogen-activated protein kinase kinase kinase 20
*Mbd1*: Methyl-CpG binding domain protein 1
*Mbl2*: Mannose-binding lectin (protein C) 2
*Mcm5*: Minichromosome maintenance complex component 5
*Meg3*: Maternally expressed 3
*Mgmt*: O-6-methylguanine-DNA methyltransferase
*miR 17–92 cluster*: MicroRNA 17–92 cluster
*Mmp7*: Matrix metallopeptidase 7
*Mmp10*: Matrix metallopeptidase 10
*Moxd1*: Monooxygenase, DBH-like 1
*Mybl*1: MYB proto-oncogene like 1
*Mybl2*: MYB proto-oncogene like 2
Mycn: MYCN proto-oncogene, bHLH transcription factor
Ncapg2: Non-SMC condensin II complex, subunit G2
*Nebl*: Nebulette
*Nisch*: Nischarin
*Nolc1*: Nucleolar and coiled-body phosphoprotein 1
*Nos1*: Nitric oxide synthase 1
*Nr2c2*: Nuclear receptor subfamily 2, group C, member 2
*Nsl1*: NSL1, MIS12 kinetochore complex component
*Nt5dc2*: 5'-Nucleotidase domain containing 2
*Parpbp*: PARP1 binding protein
*Pcna*: Proliferating cell nuclear antigen
*Pgap2*: Post-GPI attachment to proteins 2
*Phlda3*: Pleckstrin homology-like domain, family A, member 3
*Pierce1*: Piercer of microtubule wall 1
*Pkp2*: Plakophilin 2
*Plaat3*: Phospholipase A and acyltransferase 3
*Plekha2*: Pleckstrin homology domain-containing, family A (phosphoinositide binding specific) member 2
*Plk2*: Polo-like kinase 2
*Pln*: Phospholamban
*Pnliprp 1*: Pancreatic lipase related protein 1
*Pola1*: Polymerase (DNA directed), alpha 1
*Ppara*: Peroxisome proliferator activated receptor alpha
*Pparg*: Peroxisome proliferator-activated receptor gamma
*Pqlc3*: PQ loop repeat containing
*Prkd2*: Protein kinase D2
*Prdm15*: PR domain containing 15
*Prom1*: Prominin 1
*Pttg1*: PTTG1 regulator of sister chromatid separation, securin
*Rasal2*: RAS protein activator like 2
*Retnlb*: Resistin like beta
*Rorc*: RAR-related orphan receptor gamma
*Rps27*: Ribosomal protein S27
rxr: Nuclear receptor
*Samd4a*: Sterile alpha motif domain containing 4A
*Siva1*: SIVA1, apoptosis-inducing factor
*Slc7a1*: Solute carrier family 7 member 1
*Slc25a32*: Solute carrier family 25, member 32
*Slc30a2*: Solute carrier family 30 (zinc transporter), member 2
*Snx6*: Sorting nexin 6
*Sox2*: SRY-box transcription factor 2
*Spp1*: Secreted phosphoprotein 1
*Srprb*: Signal recognition particle receptor, B subunit
*Sry*: Sex determining region Y
*Sst*: Somatostatin
*Stmn1*: Stathmin 1
*Stmn3*: Stathmin 3
*Sycn*: Syncollin
*Tacc3*: Transforming, acidic coiled-coil containing protein 3
*Tagln2*: Transgelin 2
*Tead1*: TEA domain family member 1
*Tgfb1*: Transforming growth factor, beta 1
*Tk1*: Thymidine kinase 1
*Tmem98*: Transmembrane protein 98
*Tmem181c-ps*: Transmembrane protein 181C, pseudogene
*Tmem268*: Transmembrane protein 268
*Tmsb10*: Thymosin, beta 10
*Tn*c: Tenascin C
*Tnf*: Tumor necrosis factor
*Tnfrsf1b*: Tumor necrosis factor receptor superfamily, member 1b
*Top2a*: Topoisomerase (DNA) II alpha
*Tspan13*: Tetraspanin 13
*Tuba1a*: Tubulin, alpha 1A
*Tubb4b*: Tubulin, beta 4B class Ivb
*Tulp2*: Tubby-like protein 2
*Ube2c*: Ubiquitin-conjugating enzyme E2C
*Ube2t*: Ubiquitin-conjugating enzyme E2T
*Wif1*: Wnt inhibitory factor 1
*Wt1*: WT1 transcription factor
*Wwtr1*: WW domain containing transcription regulator 1
*Zcchc12*: Zinc finger, CCHC domain containing 12
*Zdhhc14*: Zinc finger, DHHC-type palmitoyltransferase 14
*Zeb2*: Zinc finger E-box binding homeobox 2
*Zfp54*: Zinc finger protein 54
Zfp472: Zinc finger protein 472
*Zfp750*: Zinc finger protein 750
*Zfp958*: Zinc finger protein 958
*Zkscan14*: Zinc finger with KRAB and SCAN domains 14
